# Response of recurrent BRAFV600E mutated ganglioglioma to Vemurafenib as single agent

**DOI:** 10.1186/s12967-014-0356-1

**Published:** 2014-12-19

**Authors:** Francesca del Bufalo, Andrea Carai, Lorenzo Figà-Talamanca, Benedetta Pettorini, Conor Mallucci, Felice Giangaspero, Manila Antonelli, Manuela Badiali, Loredana Moi, Giuseppe Bianco, Antonella Cacchione, Franco Locatelli, Elisabetta Ferretti, Angela Mastronuzzi

**Affiliations:** Department of Hematology/Oncology and Stem Cell Transplantation, Bambino Gesù Children’s Hospital, IRCCS, Piazza Sant’Onofrio 4, 00165 Rome, Italy; Department of Neuroscience and Neurorehabilitation, Neurosurgery Unit, Bambino Gesù Children’s Hospital, IRCCS, Piazza Sant’ Onofrio 4, 00165 Rome, Italy; Department of Radiology, Unit of Neuroradiology, Bambino Gesù Children’s Hospital, IRCCS, Piazza Sant’ Onofrio 4, 00165 Rome, Italy; Paediatric Neurosurgery Department, Alder Hey Children’s NHS Foundation Trust, Liverpool, UK; Department of Radiological, Oncological and Pathological Science, Sapienza University, Viale Regina Elena 291, 00161 Rome, Italy; Neuromed Institute, IRCCS, Via Atinense 18, 86077 Isernia, Pozzilli, IS Italy; Bone Marrow Transplantation Unit, Microcitemico Children’s Hospital, Via Jenner s/n 09121, Cagliari, Italy; Public Health, Clinic and Molecular Medicine Department, Microcitemico Children’s Hospital, Via Jenner s/n 09121, Cagliari, Italy; Pharmacy Unit, Bambino Gesù Children’s Hospital, IRCCS, Piazza Sant’Onofrio 4, 00165 Rome, Italy; University of Pavia, Strada Nuova, 27100 Pavia, Italy; Department of Experimental Medicine, Sapienza University, Viale Regina Elena 291, 00161 Rome, Italy

**Keywords:** Low Grade Glioma, Ganglioglioma, MAP Kinase pathway, BRAF V600E, Vemurafenib

## Abstract

**Background:**

Ganglioglioma (GG) and pilocytic astrocytoma (PA) represent the most frequent low-grade gliomas (LGG) occurring in paediatric age. LGGs not amenable of complete resection (CR) represent a challenging subgroup where traditional treatments often fail. Activation of the MAP Kinase (MAPK) pathway caused by the BRAFV600E mutation or the KIAA1549-BRAF fusion has been reported in pediatric GG and PA, respectively.

**Case presentation:**

We report on a case of BRAFV600E mutated cervicomedullary GG treated with standard chemotherapy and surgery. After multiple relapse, BRAF status was analyzed by immunohistochemistry and sequencing showing a BRAFV600E mutation. Treatment with Vemurafenib as single agent was started. For the first time, a radiological and clinical response was obtained after 3 months of treatment and sustained after 6 months.

**Conclusion:**

Our experience underline the importance of understanding the driver molecular alterations of LGG and suggests a role for Vemurafenib in the treatment of pediatric GG not amenable of complete surgical resection.

## Background

Ganglioglioma (GG) and pilocytic astrocytoma (PA) represent the most frequent low-grade gliomas (LGG) occurring in paediatric age. When complete resection (CR) is obtained, the prognosis of these tumours is excellent. If CR is not safely achievable, the management can be extremely challenging and often ineffective despite chemo and/or radiotherapy, leading to a worse prognosis.

Activation of the MAP Kinase (MAPK) pathway has been shown to be the main molecular alteration present in LGG and can be caused by duplication or mutation of the BRAF gene [1]. In PA the most frequent genetic alteration consists in a duplication of the 7q34 region leading to a KIAA1549-BRAF fusion protein that is constitutively active whereas in GG the BRAFV600E mutation is more frequent. Inhibitors of MAPK pathway have been considered as a potential target therapy for these tumours [2,3]. Among such inhibitors Vemurafenib, a competitive small molecule that selectively recognizes the ATP binding domain of the BRAFV600E mutant, has proved effective in the treatment of metastatic melanoma, a neoplasm frequently mutated for BRAF. More recently, an activity of this drug was proved also in pediatric BRAFV600E mutated malignant astrocytomas [4-6].

Herein, we report on a case of BRAFV600E mutated cervicomedullary LGG successfully treated with Vemurafenib as single agent after failure of conventional treatment.

## Case report

**Figure 1:**
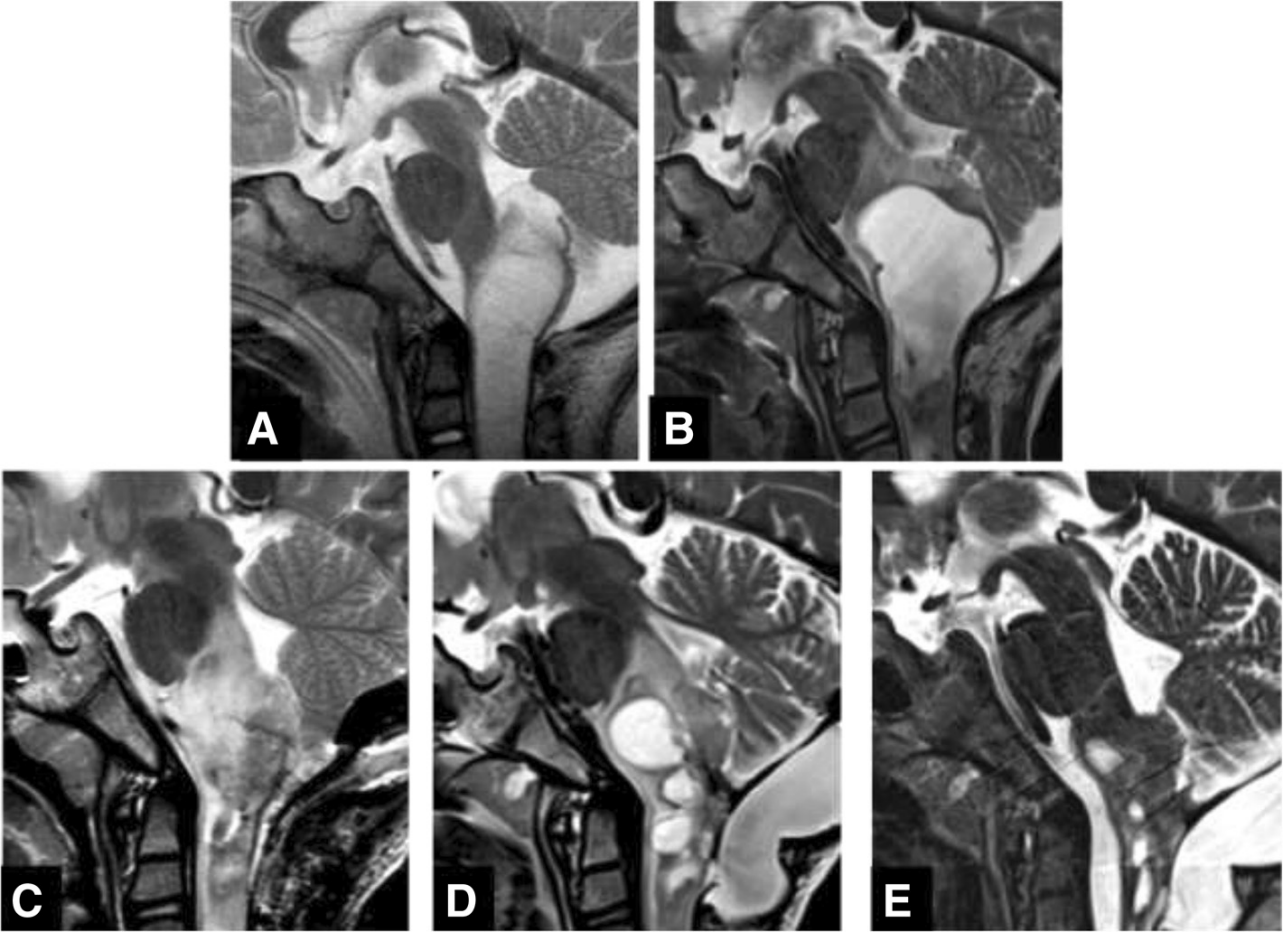
**Serial MRI features of the lesion.** Sagittal T2 weighted images show, at onset, a bulky mass extending from medulla into cervical spinal cord, dislocating the pons, the floor of the IV ventricle, the cerebellar vermis and tonsils **(A)**; increased size of both cystic and solid component of the lesion after surgical decompression and chemotherapy **(B)**; a new disease progression three months after second surgery **(C)**; further increase of cystic components **(D)**; a relevant reduction in size of both the solid and the cystic components of the lesion six months after the start of treatment **(E)**.

**Figure 2:**
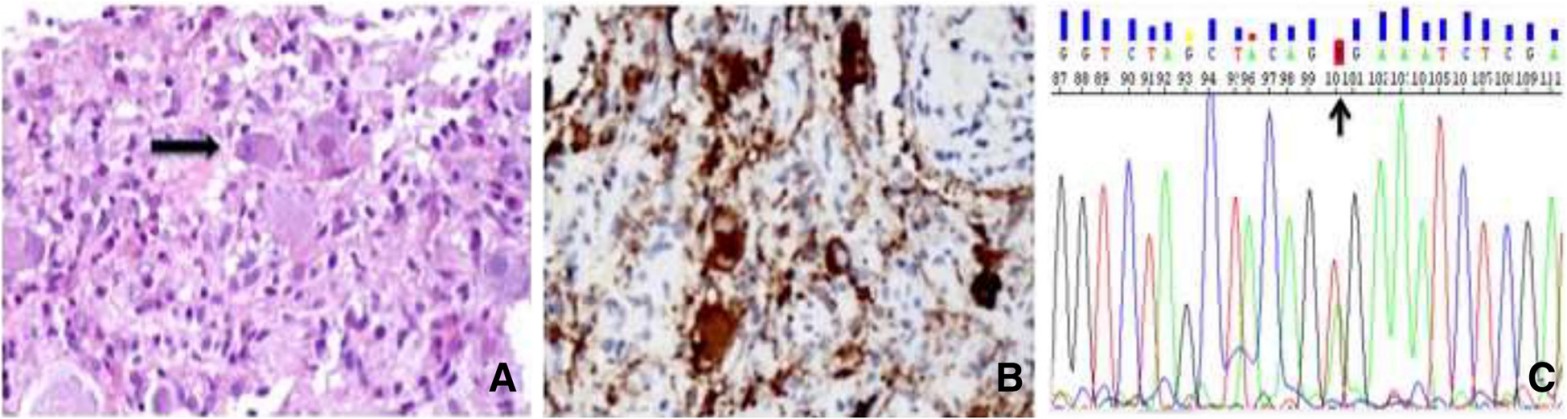
**Tumor histology at second biopsy. (A)** At the second biopsy, the neoplasm showed the presence of clusters of mature ganglion cells (arrow) in the mist of bland astrocytic cells. **(B)** The ganglion cells showed strong immunoreactivity for synaptophysin **(C)**. Electropherogram illustrate BRAF V600E (GTG/GAG) mutation detection (arrow) in tumor DNA derived from formalin-fixed paraffin-embedded specimens.

A 28-month-old boy was transferred to our emergency department from a local hospital in assisted ventilation for a respiratory insufficiency in June 2009. MRI performed during diagnostic work up revealed a bulky mass with cystic component extending from medulla into cervical spinal cord to C5 and dislocating the pons, the floor of the IV ventricle, the cerebellar vermis and tonsils (Figure 1A). As gross total resection (GTR) was not considered feasible, surgical decompression and a biopsy of the exophitic portion of the lesion were performed revealing a LGG with features compatible with PA. Polysomnographic exam revealed a relevant number of episodes of oxygen desaturation >4% of central origin. Tracheotomy was performed and chemotherapy according to the SIOP LGG 2004 protocol started. Unfortunately, the tumour did not respond to treatment showing a gradual clinical and radiological progression with worsening of the nocturnal episodes of desaturation and progressive increase of size of both a cystic portion of the lesion and the solid component (Figure 1B). A second surgery was performed in 2012 in order to reduce the cystic component of the lesion. The histological examination of the residual lesion showed the presence, in addition to the glial component, of mature ganglion cells, leading to a diagnosis of ganglioglioma (GG) (Figure 2) with classical morphology, i.e. neoplastic astrocytes and ganglion cells with dysplastic, binucleated neuron, embedded in tissue with eosinhophilic granular body and lymphocytic intratumoral infiltrate. MRI 3 months after surgery revealed a new disease progression with evidence of multicystic component in the brainstem and cervical spine, which appeared to be related to syringobulbia and syringomyelia secondary to cerebrospinal fluid outflow impairment (Figure 1C). In order to improve local control of the cystic component, a new attempt of debulking was performed; intraoperative brainstem monitoring showed functional responses in the context of the solid component of the tumour and further resection was then abandoned. Two syringe-subarachnoid stents were then inserted to achieve decompression of the cysts. Unfortunately, after an initial stabilization, slow clinical and radiological progression were documented (Figure 1D) and the child began to experience swallowing difficulties and worsening of nocturnal oxygen desaturations. Radiotherapy was not advised due to patient’s age and proton beam therapy was not deemed feasible due to extension of disease in a critical location.

Considering the progressive clinical deterioration of the patient and the absence of other effective options, molecular testing for evaluation of a target therapy was performed on the tumour tissue from the first biopsy: according to data from the literature, the KIAA-BRAF fusion gene detection and BRAFV600E testing were performed on fresh frozen (FF) tumor tissue by RT-PCR, PCR amplification and subsequent sequencing.

DNA was extracted from FF tissue specimen using the QIAamp DNA Mini Kit, as described by the manufacturer (Qiagen S.A., Courtaboeuf France). Total RNA was extracted from FF tissue using Eurogold Trifast (by Euroclone). DNA and RNA concentrations were quantified using the Nanodrop ND-1000 UV–vis spectrophotometer (Labtech France, Palaiseau, France) and the integrity of nucleic acid was determinated using Quanti-it RNA Assay kit and quanti-dsDNA BR assay kit with Quibit fluorometer (by Invitrogen- Life Technologies). Final products were stored at −20°C until use.

Moreover standard diagnostic procedure were performed. Sections were stained with ematoxilin and eosin, and immunohistochemical stain for synaptophysin (Mouse Monoclonal Antibody Synaptophysin diluition 1:200, Novocastra Clone 27G12), was performed on paraffin section using labelled strepavidin-biotin peroxidise technique. Antigen retrieval was effected by pressure cooking in citrate buffer pH6. The sections was counterstained with hematoxilin.

### *KIAA1549:BRAF* fusion-gene by sequencing

Reverse-transcription polymerase chain reaction (RT-PCR) was performed on 1 μg of total RNA using High Capacity cDNA Reverse Transcriptionkit (Life Technologies) according to the manufacturer’s protocol. The integrity of the resulting cDNA was checked by amplifying the wild-type locus of the *BRAF* gene (in exon 6 / 7) and then submitted to PCR with specific pairs of primers flanking the fusion point between the *KIAA1549* (in exon 15 or 16) and *BRAF* (in exon 9 or 11) genes as described by Jones et al. [7]. The purified PCR products were then sequenced using the BigDye Terminator v1.1 Cycle Sequencing Kit (Applied Biosystems, Courtaboeuf, France) with the forward and reverse primer used to perform the PCR. Sequencing was performed using the ABI 3130 XL DNA analyser (Applied Biosystem). The sequences of primers used were as follows: *KIAA1549* exon 15: 5′-CGG AAA CAC CAG GTC AAC GG-3′; *KIAA1549* exon 16: 5′-AAA CAG CAC CCC TTC CCA GG-3′; *BRAF* exon 9: 5′-CTC CAT CAC CAC GAA ATC CTT G-3′; *BRAF* exon 11: 5′-GTT CCA AAT GAT CCA GAT CCA TTC-3′. RT-PCR from RNA didn’t show the presence of the KIAA1549-BRAF fusion gene (data not shown).

### *BRAFV600E* mutation analysis

Mutational analysis was performed amplifying DNA with the primers as follows: *BRAF* exon 15, 5′- TCA TAA TGC TTG CTC TGA TAG GA-3′ (sense) and 5′-GGC CAA AAA TTT AAT CAG TGG A-3′ (antisense). The PCR products were purified using the automated system Biomek NXp by Beckman Coulter and Agentcourt AMPure XP reagents. Purified products were submitted to PCR cycle sequencing conditions as follow: denaturation at 95°C for 30 s, annealing at 50°C for 15 s, and extention at 60°C for 240 s. The cycle sequencing products were purified using the same automated system and Agentcourt Clean SEQ reagents. Sequencing analysis was performed using the ABI 3130 XL DNA analyser (Applied Biosystem). DNA analysis sequencing revealed BRAFV600E mutation (Figure 2C).

Based on these results, a treatment with Vemurafenib was started on compassionate use in November 2013 (240 mg, 370 mg/m^2^, twice a day (BID), equivalent to the minimal dose that proved active in the adult cohort). The therapy was overall well tolerated: accurate dermatological and ECG monitoring were performed and no ECG changes nor skin lesions were observed. The only side effect reported was a transient grade 3 Common Toxicity Criteria (version 4) skin rash that resolved spontaneously. MRI performed 3 months after the start of treatment revealed, for the first time, a reduction in size of both the solid and the cystic components of the disease, a trend confirmed after 6 months of treatment (Figure 1E). Accordingly, clinical symptoms improved with complete restoration of the swallowing function and reduction of the nocturnal episodes of desaturation.

## Discussion

Gangliogliomas are rare, well-differentiated, neuroepithelial tumors that most commonly affect children and young adults. They occur more commonly in the supratentorial region, mostly in the temporal lobe (up to 85%), but can occasionally develop also in the brainstem, cerebellopontine angle, thalamus, optic nerve and spinal cord. Included in the broad category of LGG, they are considered indolent tumors with excellent long-term survival [8].

Surgery is generally recognized as the treatment of choice for GGs, aimed at achieving a safe complete tumour resection [9]. Accordingly, the location of the tumour has also an impact on the PFS, influencing the management of the disease and the possibility of achieving a radical surgery [10]. On these bases, LGGs occurring along the midline (chiasma/hypothalamus, basal ganglia and brainstem) display a poorer outcome as compared to tumours in other locations, with a higher risk of disease progression and an indolent course, resulting in a high OS [11]. The role of chemotherapy in the treatment of LGG is still debated: several approaches have been evaluated showing variable response rates with substantially low 5-years PSF [12]. Despite these results, to date it represents the only available approach to delay RT in younger children with unresectable LGG.

Radiotherapy (RT) is considered the treatment of choice for LGG not amenable of surgical resection, therefore representing the best option for centrally located tumours [13,14]. Unfortunately, adverse effects preclude its use in younger children (until at least 5, possibly 8 years of age), leading to a substantial increase in the risk of progression for this category of patients. Moreover, even when used in older children, long term vasculopathy, hearing loss and neurocognitive and endocrinological sequelae remain a relevant concern [15]. Therefore, taking into consideration the natural history of tumour stabilization, its indolent course and the high likelihood of long-term survival, the use of RT must be carefully weighed.

Our child presented with a rare cervicomedullary GG. Although the histology resulted favorable, the location and the age of the child represented relevant negative prognostic factors, preventing complete surgical removal of the lesion and the use of RT. In order to obtain a stabilization of the disease, standard chemotherapy based on SIOP LGG 2004 protocol was administered. Unfortunately, but not surprisingly, the child clinically and radiologically progressed at the end of the treatment, confirming the indolent but progressive course of this disease.

The recent finding of driver genomic alterations in BRAF gene in LGG and the development of new molecules that interfere with this deregulated signaling are highly attractive, especially in patients with midline, unresectable tumours, and when RT is not recommended, in order to overcome treatment limitations and improve cure rate.

In 2008, different groups identified gains at 7q34 of approximately 2 megabases in size in most LGG, representing segmental duplications of the region [1,2,16-18]. This duplication leads to the formation of a fusion between the KIAA1549 locus and BRAF and the resulting protein displays a constitutively activated kinase activity causing an aberrant activation of the downstream MAPK/ERK pathway. Subsequent studies revealed other, less common, molecular alterations in BRAF gene driving activation of the same pathway [3,19,20]: the most frequent is the point mutation that occurs at codon 600 (BRAFV600E), firstly associated with several non-CNS human tumors, that results in substitution of valine by glutamic acid [21,22].

BRAFV600E mutation appears to be particularly associated with paediatric GG where its status changes based on the anatomical location. Although Schindler et al. could not identify it, the mutation seems to be present in a relatively high percentage of cases with brainstem location [3,23-25].

The prognostic relevance of BRAF duplication/mutation is not clear yet: some reports suggest an association with a better outcome in children displaying BRAF fusion and a trend toward a lower PFS in LGG expressing BRAFV600E mutation while other groups could not confirm these findings [26-30]. Dahiya et al. revealed a significantly worse recurrence-free survival of BRAFV600E-mutated GG compared to negative tumors, suggesting a negative prognostic role for this mutation in GG [8].

Novel therapies targeting the altered BRAF pathway have been developed, including the oral drug Vemurafenib. After showing impressive, although transient, results on recurrent melanoma, it has been approved by the FDA for the treatment of unresectable or metastatic melanoma. The drug proved well tolerated so far in adults, with arthralgia, rash, alopecia, fatigue, photosensitivity reaction, nausea, pruritus and skin lesions reported as main toxicities [31]. Overall, a variety of skin toxicities has been reported and therefore a careful examination is recommended during treatment [32].

The use of an oral target therapy to control the disease in children with unresectable LGG is highly suitable. *In vitro* and *in vivo* studies of paediatric astrocytoma cell lines expressing BRAFV600E mutation have been performed and show that target inhibition of mutated BRAF exerts an antiproliferative activity and slows tumour growth, improving survival [4]. With this strong supportive rationale, a safety and pilot efficacy clinical trial of Vemurafenib against BRAFV600E mutant recurrent or refractory LGG in children has recently started (ClinicalTrials.gov Identifier: NCT01748149). To the best of our knowledge, only one report on the use of this drug in pediatric low grade GG has been published and showed encouraging results, in association with vinorelbine [33].

Taking into consideration all these evidences and the persistent progression of our patient, we decided to evaluate the presence of BRAFV600E mutation in order to initiate treatment with Vemurafenib. Since no pharmacokinetic data nor toxicity analysis are available in the pediatric population to date, we decided to start the treatment with the minimal dose that proved active in the adult cohort, and maintained it in consideration of the results shown [34]. The excellent, rapid and sustained response documented in our child after 6 months of treatment shows a relevant efficacy of this small-molecule inhibitor in a challenging subcategory of LGG, although a longer follow-up is required to define the long-term response to this drug. Notably, our patient did not receive any other concurrent chemotherapy, proving that the observed response can be attributed exclusively to the BRAF inhibitor.

The lesson provided by the use of Vemurafenib in melanoma patients, however, warns treatment [35]. Moreover, in the context of malignant GG, few reports have proved a not uniform activity of Vemurafenib [6]. Although not clear yet, these diverse responses are likely related to the complex genetic aberrations present in malignant gliomas which might induce the overactivation of MAP kinase through alternative pathways, regardless of the BRAF status, and thus impair the efficacy of the treatment. Similarly, the mechanisms underlying the acquired resistance are multiple and not fully understood yet. Most of them rely upon the alternative reactivation of the MAP kinase signaling pathway through the mutational activation of other key molecule of the pathway, such as NRAS, MEK1 or MEK2, or the occurrence of BRAF-V600E splice variants [36-38]. Moreover, MAPK pathway-independent mechanisms of resistance have been also suggested, involving alterations that lead to the upregulation of the PI3K-AKT signaling pathway [39]. Therefore, the combination with either MEK, ERK or PI3K inhibitors might be considered to overcome both intrinsic and acquired resistance.

The optimal tolerance to the treatment and the advantage of the oral administration represent relevant aspects in the context of LGG as they reduce the burden of the frequent hospitalization that these children and their families face, sometimes for several years. It is important to point out, however, that the induction of secondary cutaneous lesions and the promotion of proliferation of pre-malignant cells harboring RAS mutation in non-cutaneous tissues, reported in some adult patients, raise considerable concern, especially in the pediatric population. Therefore, a careful case-specific consideration of the risk/benefit ratio is mandatory until more detailed documentation will be provided by clinical trials.

## Conclusions

To our knowledge, this is the first case describing the use of Vemurafenib as single agent in paediatric GG. Our experience, although limited to a case report, and the review of the literature underline the importance of understanding the driver molecular alterations of LGG to improve treatment strategies, through target therapies, and ultimately outcome of these patients. As specific BRAF inhibitors are now available, the evaluation of BRAF status in children with tumors not amenable of GTR should be considered in order to offer a valuable therapeutic alternative. A wider molecular signature, moreover, might be required in case of low response or relapse, in order to further improve the activity by multiple targeting. Large clinical trials are needed to further evaluate the pharmacokinetic profile, safety and efficacy of BRAF inhibitor in the treatment of LGG with this signature and the time of suspension of this therapy, considering the possibility of relapse/progression of disease at the end of treatment.

## Consent

Written informed consent was obtained from the patient’s parent for the publication of this report and any accompanying images.
